# Evidence of Increased Oxidative Stress in the Placental Tissue of Women Who Suffered an Episode of Psychosis during Pregnancy

**DOI:** 10.3390/antiox12010179

**Published:** 2023-01-12

**Authors:** Miguel A. Ortega, Oscar Fraile-Martinez, Cielo García-Montero, Sonia Rodriguez-Martín, Rosa M. Funes Moñux, Coral Bravo, Juan A. De Leon-Luis, Jose V. Saz, Miguel A. Saez, Luis G. Guijarro, Guillermo Lahera, Jorge Monserrat, Fernando Mora, Javier Quintero, Julia Bujan, Natalio García-Honduvilla, Melchor Alvarez-Mon, Miguel Angel Alvarez-Mon

**Affiliations:** 1 Department of Medicine and Medical Specialities, Faculty of Medicine and Health Sciences, University of Alcalá, 28801 Alcalá de Henares, Spain; 2 Ramón y Cajal Institute of Sanitary Research (IRYCIS), 28034 Madrid, Spain; 3 Service of Pediatric, Hospital Universitario Principe de Asturias, 28801 Alcalá de Henares, Spain; 4 Department of Public and Maternal and Child Health, School of Medicine, Complutense University of Madrid, 28040 Madrid, Spain; 5 Department of Obstetrics and Gynecology, University Hospital Gregorio Marañón, 28009 Madrid, Spain; 6 Health Research Institute Gregorio Marañón, 28009 Madrid, Spain; 7 Department of Biomedicine and Biotechnology, Faculty of Medicine and Health Sciences, University of Alcalá, 28801 Alcala de Henares, Spain; 8 Pathological Anatomy Service, Central University Hospital of Defense-UAH Madrid, 28801 Alcala de Henares, Spain; 9 Unit of Biochemistry and Molecular Biology (CIBEREHD), Department of System Biology, University of Alcalá, 28801 Alcalá de Henares, Spain; 10 Psychiatry Service, Center for Biomedical Research in the Mental Health Network, University Hospital Príncipe de Asturias, 28806 Alcalá de Henares, Spain; 11 Department of Psychiatry and Mental Health, Hospital Universitario Infanta Leonor, 28031 Madrid, Spain; 12 Department of Legal Medicine and Psychiatry, Complutense University, 28040 Madrid, Spain; 13 Immune System Diseases-Rheumatology and Internal Medicine Service, University Hospital Príncipe de Asturias, CIBEREHD, 28806 Alcalá de Henares, Spain

**Keywords:** psychosis during pregnancy, oxidative stress, placenta, maternofetal well-being, NADPH oxidases (NOX), nitric oxide synthases (NOS), poly (ADP-ribose) polymerase (PARP)

## Abstract

Psychosis is a complex clinical syndrome resulting in a loss of contact with reality and alterations in behavior and sensorial and motor functions. Although the onset of psychosis can be related to any medical condition, most cases of psychosis are not fully understood. Psychosis may manifest for the first time during pregnancy, which is detrimental to maternofetal well-being. The impact of having a first episode of psychosis during pregnancy on the placenta has not yet been explored. Oxidative stress is thought to take part in the etiopathogenesis of this complex disorder, and this condition can also affect the placenta as it is highly sensitive to changes in the maternal environment. In this sense, the aim of the present work was to study the gene and protein expression through RT–qPCR and immunohistochemistry, respectively, of oxidative stress markers (NOX-1, NOX-2, iNOS, eNOS and PARP) in the placental tissue of women who underwent a first episode of psychosis during pregnancy (FE-PW) in comparison to healthy pregnant women. Our results showed augmented gene and protein expression of NOX-1, NOX-2, iNOS and PARP in the placental tissue of FE-PW. For the first time, we demonstrated that oxidative stress may have an important pathophysiological role in this tissue, aiding in explaining the impact of psychosis on pregnancy and the need for future studies in this field to guide better clinical management of these patients.

## 1. Introduction

Psychosis is an amalgamation of psychological symptoms resulting in a loss of contact with reality [1]. Although a formal definition of psychosis is not given by the Diagnostic and Statistical Manual fifth edition (DSM-5), it does characterize psychotic disorders by alterations in at least one of the five following domains: delusions, hallucinations, disorganized thought, grossly disorganized or abnormal motor behavior (including catatonia) and negative symptoms [2,3]. It has been recognized that psychotic disorders can be either primary (if symptomatic of a psychiatric disorder) or secondary (as a consequence of any medical condition, including illicit drug use) [4]. The etiological and pathophysiological bases of psychotic disorders remain poorly understood. It is thought that its different manifestations are related to changes in particular neurotransmitters, such as dopamine, glutamate, gamma-amino-butyric acid (GABA) or acetylcholine [1]. However, compelling evidence supports that all of these neurotransmitter changes are a consequence rather than a cause, and all psychotic symptoms are common outcomes of a spectrum of different causes and etiopathogenetic pathways that converge to produce a similar clinical picture [5].

Pregnancy is a period in which a woman’s body undergoes a plethora of systemic changes affecting virtually all tissues and organs [6]. Despite being highly unlikely, a first episode of psychosis can appear in pregnancy, although there are few notions about the etiopathogenic mechanisms relating pregnancy to psychosis. The main risk factors described include a family history of psychosis or women with a preexisting or undiagnosed psychotic/mood disorder [7]. Likewise, stressful conditions in this period, including phenomena of obstetric violence, can drive to the onset of psychosis in susceptible women [8,9,10,11,12]. Past works have found that pregnant women with psychosis present an elevated risk of multiple obstetric and neonatal complications, including antepartum/postpartum hemorrhage, C-section or premature delivery, fetal abnormalities, fetal distress and/or poor fetal growth and stillbirth, among others [13]. Likewise, suffering from a first episode of primary psychosis during pregnancy can also have long-term consequences for the mother, as it has been established that women with a history of psychotic disorder are at high risk of suffering from a psychiatric illness in the future [14]. Alterations in placental structure and function may be partly responsible for the adverse outcomes related to psychosis, as previous works have established that this organ undergoes significant changes in women with psychiatric disorders during pregnancy [15,16,17]. In turn, these changes can have a significant impact in the offspring, affecting neurodevelopment and increasing the risk of suffering from different neuropsychiatric disorders [18]. Thus, analyzing the placental tissue after a first episode of psychosis in pregnancy could help to understand and evaluate the impact of this condition in the maternofoetal wellbeing.

Oxidative stress (OS) is caused by an imbalance between free radicals and antioxidants, representing a potential etiopathological mechanism in different conditions, including psychosis [19,20,21]. In addition, previous works have shown that patients with a first episode of psychosis present a reduced antioxidant status [22], suggesting that OS is closely linked with the onset and development of this condition. In this sense, previous works have established that the placenta is highly sensitive to the maternal environment and that increased OS markers are feasible indicators of maternofoetal suffering in the context of various obstetric complications, such as preeclampsia, fetal-growth restriction and gestational venous hypertension [23]. 

Several blood and tissue markers have been studied to define the extent of OS in the placenta. For instance, overexpression of NADPH oxidases 1 and 2 (NOX-1/NOX-2) represents an important source of a group of free radicals designated reactive oxygen species (ROS), which have been suggested as potential OS markers [24]. Similarly, nitric oxide synthases (NOS) are involved in the generation of free radicals, particularly reactive nitrogen species (RNS). There are three main types of NOS: endothelial NOS (eNOS) and inducible NOS (iNOS), which are expressed in different tissues and organs, including the placenta, and neuronal NOS (nNOS), which is restricted to nervous structures and other tissues and is not expressed in human placenta [25]. As with NOX, overexpression of eNOS and iNOS has been related to different complications in pregnancy [26]. Finally, poly (ADP-ribose) polymerase (PARP) is an enzyme involved in DNA repair, and its overexpression has been reported under OS conditions to counteract the oxidative damage to this structure [27]. 

Hence, the aim of the present work was to establish whether enhanced OS can be observed in the placental tissue of pregnant women who suffer a first episode of psychosis (FE-PW) in comparison to nonpathological controls (HC-PW). To achieve this goal, the gene and protein expression of NOX-1, NOX-2, iNOS, eNOS and PARP were analyzed using real-time quantitative PCR (RT–qPCR) and immunohistochemistry, respectively.

## 2. Patients and Methods

### 2.1. Study Design

This was an observational, analytical and prospective study of 42 women in the third trimester of pregnancy; 22 of them were clinically diagnosed with a first episode of psychosis (FE-PW), and 20 of them had no complications during pregnancy (HC-PW). The inclusion criteria were (1) a psychiatrist-confirmed diagnosis of FE-PW following the DSM-5 criteria, using the Structured Clinical Interview for DSM-5 (SCID-5) [28]; (2) pregnant women aged 18 to 45 years; and (3) fluent Spanish speaking that enabled a proper assessment. The symptoms were assessed using the Positive and Negative Syndrome Scale (PANSS) [29]. Exclusion criteria were: meeting the diagnostic criteria for another Axis-I mental disorder or intellectual disability; or a history of neurodevelopmental disorders or head injury with loss of consciousness. The median age in the FE-PW group was 33.5 (21–42) years, and the median gestational age was 40 (38–41) weeks. For HC-PW, the median age was 33.5 (25–39) years, and the median gestational age was 40 (39–42) weeks. The sociodemographic and clinical features of these patients are summarized in Table 1.

Before enrollment, each patient was duly informed, providing signed written consent. This study passed the Clinical Research Ethics Committee of Central University Hospital of Defense University of Alcalá (37/17), being conducted following the ethical principles of autonomy, beneficence, non-maleficence and distributive justice, as well as the norms of good clinical practice, the principles of the Declaration of Helsinki (2013) and the Oviedo Agreement (1997).

### 2.2. Tissue Samples

Postpartum placental biopsies were collected from all 42 women. For each sample, five pieces of placenta-mixed cotyledons were included and then separated into two different sterile tubes, one with minimal essential medium (MEM; Thermo Fisher Scientific, Inc., Waltham, MA, EE USA) with 1% antibiotic/antifungal (streptomycin, amphotericin B and penicillin; Thermo Fisher Scientific, Inc.) and another with RNAlater® solution (Ambion; Thermo Fisher Scientific, Inc., Waltham, MA, USA). USA). The placenta samples were processed in a sterile environment in a class-II laminar flow hood (Telstar AV 30/70 Müller 220 V 50 MHz; Telstar; Azbil Corporation, Tokyo, Japan).

The samples placed in 1 ml of RNAlater® were stored at −80 °C until processing for the gene expression study. The samples placed in MEM were washed and resuspended 5 times in MEM free of antibiotics to remove any erythrocytes. Subsequently, each sample was cut with a scalpel into 2 cm fragments and then fixed in F13 (60% ethanol, 20% methanol, 7% polyethylene glycol and 13% distilled water) following established protocols [30]. Afterward, paraffin-embedded samples were formed using molds. Once the paraffin solidified, an HM 350 S rotary microtome (Thermo Fisher Scientific, Inc., Waltham, MA, USA) was used to obtain 5 µm thick sections, which were placed in a hot water bath and collected into glass slides treated with 10% polylysine to facilitate the adherence of the sections. Finally, these samples were subjected to immunohistochemical techniques.

### 2.3. Gene Analysis

The guanidinium thiocyanate-phenol–chloroform method was used to perform RNA extraction as described in previous works [31]. This method allows the analysis of the mRNA expression levels of studied genes. Complementary DNA (cDNA) was synthesized by reverse transcription (RT) reaction from 50 ng/µL RNA samples. A total of 4 µL of each sample was mixed with 4 µL of 0.25 µg/µL oligo-dT solution (Thermo Fisher Scientific, Inc., Waltham, MA, USA) and then placed at 65 °C for 10 min in a dry bath (AccuBlock, Labnet International Inc., New Jersey, USA), thus achieving RNA denaturation. Then, the samples were placed on ice and mixed with 10 µL of RT mix with the following products: 2.8 µL of First Strand Buffer 5X (250 mM Tris-HCl and pH 8.3; 375 mM KCl:15 mM MgCl_2_) (Thermo Fisher Scientific, Inc., Waltham, MA, USA); 1 µL of RT enzyme (all from Thermo Fisher Scientific, Inc., Waltham, MA, USA); 2 µL of 10 mM deoxyribonucleotide triphosphate; 2 µL of 0.1 M dithiothreitol; 1.7 µL of DNase- and RNase-free water and 0.5 µL of RNase inhibitor (RNase Out). RT was performed in a G-Storm GS1 thermocycler (G-Storm Ltd.). The samples were incubated at 37 °C for 75 min to allow cDNA synthesis. Then, the temperature was increased to 70 °C over 15 min in order to allow reverse transcriptase denaturation. Subsequently, the temperature was gradually reduced to 4 °C. Negative RT was performed to ensure the lack of genomic DNA contamination in the RNA samples, in which the M-MLV reverse transcriptase was replaced with DNase- and RNase-free water. cDNA produced at room temperature was diluted in DNase- and RNase-free water (1:20) and kept at −20 °C until further use.

Specific primers for the selected genes are listed in Table 2. Their design was performed through Primer-BLAST and AutoDimer online applications [32,33]. We used the TATA-box binding protein (TBP) gene as a control to normalize our results, as this product is constitutively expressed [34]. Gene expression units are expressed as relative quantities of mRNA. RT–qPCR was performed on a StepOnePlus™ System (Applied Biosystems; Thermo Fisher Scientific, Inc.) using the relative standard curve method. Details about the reaction performed are summarized in [35].

### 2.4. Protein Studies

An avidin–biotin complex with avidin peroxidase was used for antigen/antibody reaction detection following established protocols [36]. Immunohistochemical studies were performed on placenta samples embedded in paraffin. The antibodies employed in our study are detailed in the protocol specifications (Table 3).

Placental tissues were washed three times with 1x PBS for 5 minutes. Then, after blocking nonspecific binding sites with 3% bovine serum albumin (BSA) diluted in PBS at room temperature for 30 minutes, samples were incubated with the primary antibody for 90 min and subsequently with 3% BSA Blocker (cat. no. 37,525; Thermo Fisher Scientific, Inc., Waltham, MA, USA) and PBS (4 °C overnight). The following day, placental samples were incubated with biotin-conjugated secondary antibody diluted in PBS for 1.5h at room temperature (Table 3). Subsequently, an avidin–peroxidase conjugate ExtrAvidin®-Peroxidase (Sigma–Aldrich; Merck KGaA, San Luis, MO, USA) was added for one hour at room temperature (1:200 dilution in PBS). Eventually, a chromogenic diaminobenzidine (DAB) substrate kit (cat. no. SK-4100; Maravai LifeSciences, California, CA, USA) was employed to determine the protein expression level. The staining solution was prepared before exposure, mixing 5 mL of distilled water, four drops of DAB, two drops of hydrogen peroxide and two drops of buffer.

The application of the peroxidase chromogenic substrate for 15 min at room temperature facilitated the detection of a brown staining as a signal of protein expression. Negative controls for the different proteins were assigned to each section, replacing the incubation with primary antibody for PBS solution. Carazzi’s hematoxylin was used for 15 min to achieve contrast in all tissues.

### 2.5. Evaluation of Histopathological Expression and Statistical Analysis

Five different sections and ten fields were randomly examined for each patient. Immunohistochemical expression was classified as positive(+) when the mean stained area in the studied sample was equal or superior to 5% of the total, following the immunoreactive score (IRS) [37]. This method allowed us to classify immunostaining using the following scale: 0–1, minimum staining (≤25%); 2, moderate staining (25–65%) and 3–4, strong staining (≥65–100%). Two independent histologists evaluated each sample. A Zeiss Axiophot optical microscope (Carl Zeiss, Oberkochen, Germany) was used for histological determination. 

For statistical processing, the GraphPad Prism® v6.0 (GraphPad, Inc., San Diego, CA, USA) program was employed. The Mann–Whitney U test was performed to compare both groups, and the results are expressed as the median ± SD. Significant values were established as *p* < 0.05 (*), *p* < 0.01 (**) and *p* < 0.001 (***).

## 3. Results

### 3.1. Placentas of Women with a First Episode of Psychosis during Pregnancy Exhibited Increased Protein and Gene Expression of NOX-1 and NOX-2

**Figure 1:**
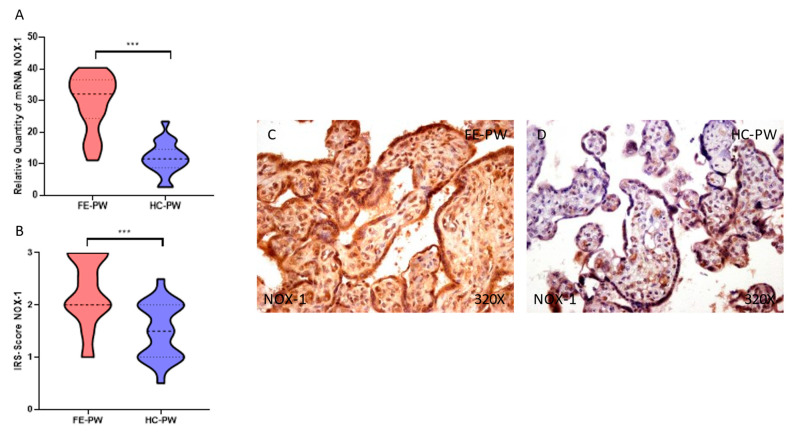
(**A**) NOX-1 mRNA expression in the FE-PW group (first episode of psychosis during pregnancy) and HC-PW group (healthy controls). (**B**) IRS scores for NOX-1 expression in the placental villi of the FE-PW group and the HC-PW group. (**C**,**D**) Images showing immunostaining for NOX-1 in the placental villi of the FE-PW group and the HC-PW group. *p* < 0.001 (***).

First, we evaluated the gene and protein expression of NOX-1 and NOX-2 by RT-qPCR and immunohistochemistry, respectively, in the placentas of women with FE-PW and compared them with the HC-PW group. We found that NOX-1 gene expression was significantly higher in the FE-PW group than HC-PW group (FE-PW = 29.385 ± 8.843 relative quantity mRNA [RQ]); HC-PW= 11.716 ± 5.058; *** *p* < 0.001, Figure 1A. Regarding protein expression, we observed a significant increase in NOX-1 expression in the placental tissue of the FE-PW group (FE-PW = 2.250 ± 0.650 versus HC-PW= 1.500 ± 0.538, *** *p* < 0.001, Figure 1B). We noted that the increased protein expression of NOX-1 occurred in the entire villous structure, including syncytiotrophoblasts, cytotrophoblasts, the matrix and fetal capillaries (Figure 1C).

**Figure 2:**
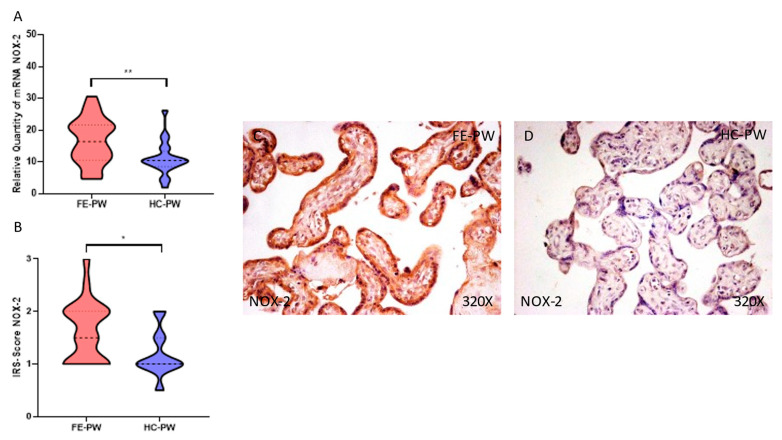
(**A**) NOX-2 mRNA expression in the FE-PW group (first episode of psychosis during pregnancy) and HC-PW group (healthy controls). (**B**) IRS scores for NOX-2 expression in the placental villi of the FE-PW group and the HC-PW group. (**C,D**) Images showing immunostaining for NOX-2 in the placental villi of the FE-PW group and the HC-PW group. *p* < 0.05 (*); *p* < 0.01 (**).

Similarly, when evaluating the gene expression of NOX-2, we observed a significant increase in the FE-PW group (FE-PW = 16.563 ± 7.287 versus HC-PW = 10.966 ± 5.538; ** *p* = 0.0046, Figure 2A). For protein expression, we found a significant increase in NOX-2 in the FE-PW group (FE-PW = 1.614 ± 0.576 versus HC-PW = 1.175 ± 0.438; *p* = 0.0111, Figure 2B). When evaluated with microscopy, augmented NOX-2 expression was observed in all of the different parts of the villi, including the syncytiotrophoblasts, cytotrophoblasts, matrix and fetal capillaries (Figure 2C).

### 3.2. Placentas of Women with a First Episode of Psychosis during Pregnancy Displayed Augmented Immunohistochemical and Gene Expression of iNOS but Normal eNOS

**Figure 3:**
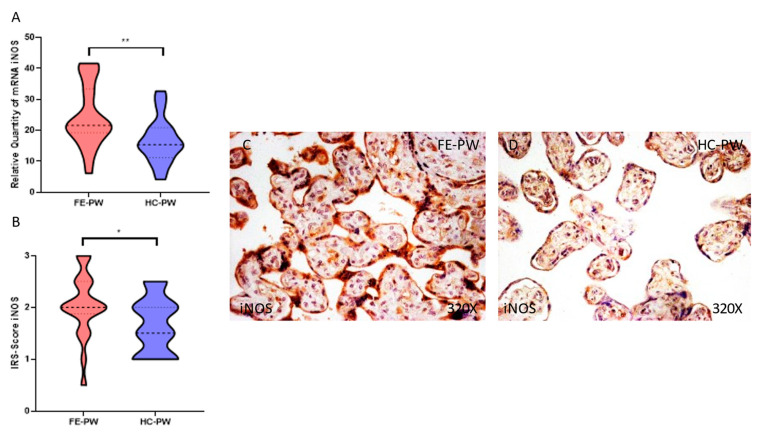
(**A**) iNOS mRNA expression in the FE-PW group (first episode of psychosis during pregnancy) and HC-PW group (healthy controls). (**B**) IRS scores for iNOS expression in the placental villi of the FE-PW group and the HC-PW group. (**C**,**D**) Images showing the immunostaining for iNOS in the placental villi of the FE-PW group and the HC-PW group. *p* < 0.05 (*); *p* < 0.01 (**).

We investigated the gene and protein expression of iNOS and eNOS by RT-qPCR and immunohistochemical studies in the placentas of women in the FE-PW and HC-PW groups. We found that iNOS gene expression was significantly higher in the FE-PW group than in the HC-PW group (FE-PW = 24.444 ± 10.157 versus HC-PW = 16.646 ± 7.332; ** *p* = 0.005, Figure 3A). Regarding protein expression, we found a significant increase in iNOS expression in the placental tissue of the FE-PW group (FE-PW = 2.045 ± 0.615 versus HC-PW = 1.650 ± 0.540; * *p* = 0.0302, Figure 3B). Expression of iNOS could be widely observed in the different cells of the placental villi of the FE-PW group (Figure 3C).

**Figure 4:**
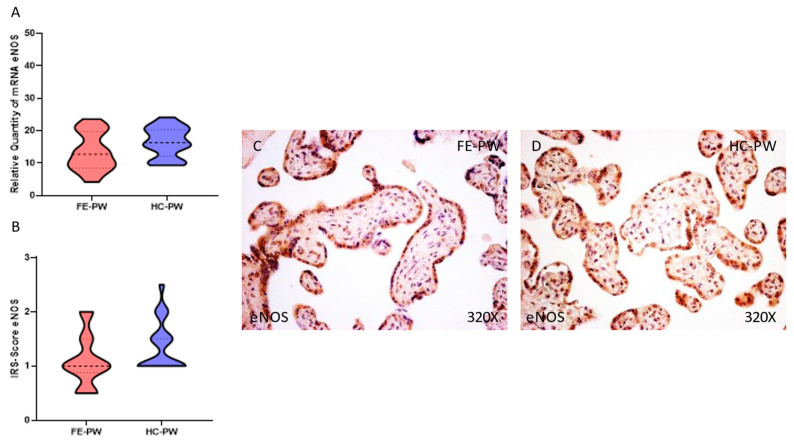
(**A**) eNOS mRNA expression in the FE-PW group (first episode of psychosis during pregnancy) and HC-PW group (healthy controls). (**B**) IRS scores for eNOS expression in the placental villi of the FE-PW group and HC-PW group. (**C**,**D**) Images showing the immunostaining for eNOS in the placental villi of the FE-PW group and HC-PW group.

However, we did not find any statistically significant difference in the expression of eNOS in the placenta of the FE-PW group relative to the HC-PW group either at the gene (FE-PW = 13.477 ± 5.770 versus HC-PW = 16.500 ± 4.601; *p* = 0.0800, Figure 4A) or protein expression level (FE-PW = 1.091 ± 0.479 versus HC-PW = 1.350 ± 0.462; *p* = 0.0713, Figure 4B). Histological observations of the placental villi in both groups did not reveal any significant difference in the protein expression of eNOS (Figure 4C).

### 3.3. Placentas of Women with a First Episode of Psychosis during Pregnancy Showed Increased Protein and Gene Expression of PARP

**Figure 5:**
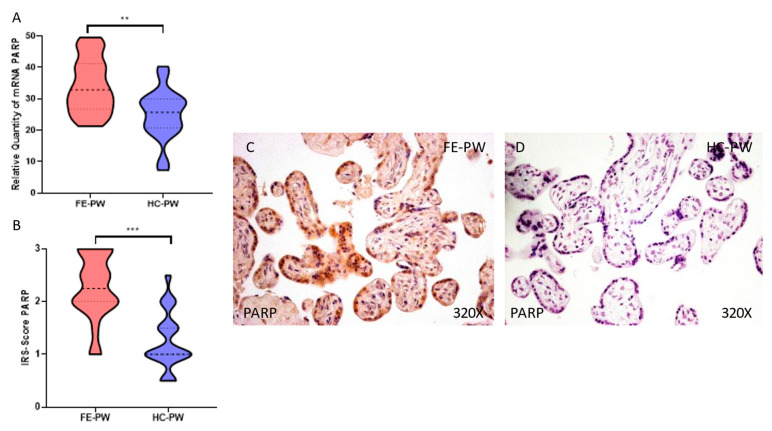
(**A**) PARP mRNA expression in the FE-PW group (first episode of psychosis during pregnancy) and HC-PW group (healthy controls). (**B**) IRS scores for PARP expression in the placental villi of the FE-PW group and the HC-PW group. (**C**,**D**) Images showing the immunostaining for PARP in the placental villi of the FE-PW group and HC-PW group; *p* < 0.01 (**); *p* < 0.001 (***).

Finally, we evaluated the gene and protein expression of PARP in the placentas of women in the FE-PW and HC-PW groups. We observed that PARP gene expression was significantly higher in the FE-PW group than in the HC-PW group (FE-PW = 34.107 ± 8.674 versus HC-PW = 25.013 ± 8.026; ** *p* = 0.0019, Figure 5A). In terms of protein expression, we found a significant increase in PARP in the placental tissue of the FE-PW group (FE-PW = 2.273 ± 0.612 versus HC-PW= 1.250 ± 0.550; *** *p* = 0.001, Figure 5B). An increased expression of PARP was observed throughout the placental villi cells (Figure 5C).

## 4. Discussion

In this work, we demonstrated increased gene and protein expression of NOX-1, NOX-2, iNOS and PARP, establishing that the placental tissue of FE-PW displayed an augmented expression of OS markers. This can help in understanding the negative consequences of undergoing a psychotic episode during pregnancy, as these placental changes may be indicators of maternofetal suffering [13,37,38].

Pregnancy is strongly linked to OS, especially under pathological conditions [39]. Recent works have shown that patients with a first episode of psychosis show a reduced antioxidant status along with a marked increase in oxidative markers [22,40]. The environment in which the mother lives exposes the placental tissue to a set of chemical, physical and biological agents of OS, which may have pathological consequences in this organ [41]. OS plays a central role in placental development and can be normally observed during nonpathological pregnancies; however, an exacerbated oxidative environment may damage cellular proteins, lipids and DNA, hence impairing placental function and interrupting maternofetal communication [42]. Frequently, OS is concomitant with hypoxic and inflammatory phenomena, forming a complex network needed to understand the underlying mechanisms of disease pathogenesis [43]. Indeed, previous works have proven the coexistence of an inflammatory environment, OS and hypoxia in the placenta of women with pathological pregnancies [30,44,45]. Hence, based on our results, further studies should be conducted to analyze inflammatory or hypoxic markers in this tissue, aiding in explaining the increased OS observed in FE-PW.

First, we found a significant increase in the enzymes NOX-1 and NOX-2. These are membrane-bound proteins involved in the transfer of electrons across the plasma membrane to molecular oxygen, eventually resulting in the generation of the superoxide anion and subsequently ROS [46]. In addition, NOX enzymes are potentially involved in the control of proliferative and intracellular signaling, acting as oxygen sensors in different tissues [47]. An augmented expression of NOX-1 and NOX-2 can be observed in the placenta during the early stages of physiological pregnancies [48]; however, an enhanced expression of both enzymes has been described in the placenta at term among women with different obstetric complications [45,49,50]. Hence, an enhanced expression of NOX-1 and NOX-2 can be an indicator of OS present in the term placenta in FE-PW, although additional studies should be aimed at evaluating systemic markers of OS in these women.

Likewise, we also demonstrated an increased expression of iNOS but not of eNOS in the placental tissue of women affected by a psychotic episode. Nitric oxide synthases are involved in the generation of nitric oxide (NO) from l-arginine. However, whereas eNOS is constitutively expressed mostly in endothelial cells and produces NO in a calcium-dependent manner, iNOS can be expressed in many cell types in response to proinflammatory cytokines or other stressors, and once expressed, it is constantly active and is not regulated by calcium [25,51]. iNOS-derived NO has been associated with the pathogenesis and progression of several diseases, but in turn, iNOS activation can modulate the metabolism to survive and cope with stress conditions [52]. Previous works have established that there may be a pathophysiological role of exacerbated iNOS expression in the placental tissue of women with preeclampsia [53]. More specifically, it seems that iNOS upregulation is critically related to enhanced apoptotic cell death triggered by endoplasmic reticulum stress, accompanied by a downregulation of eNOS. However, we did not find a reduced expression of eNOS in FE-PW.

Finally, we found an increased expression of PARP in the placenta of FE-PW in comparison to HC-PW. According to previous studies, the overexpression of PARP may be an indicator of oxidative damage of DNA, as PARP is directly implicated in sensing and repairing DNA strand breaks [54]. In addition, PARP hyperactivation appears to alter a set of cellular processes and participate in inflammatory signal transduction and the production of inflammatory mediators [55]. Not only OS but also hypoxia-inducible factor 1 α (HIF-1α) seems to activate PARP, inducing apoptosis in different tissues such as the placenta [56]. In more detail, an increased placental expression of PARP was observed after hypoxia–reoxygenation phenomena but not with sustained hypoxia alone [57], suggesting that placental malperfusion may pathologically induce the overexpression of PARP and apoptotic cell death. Conversely, other obstetric diseases characterized by a low placental expression of PARP display reduced apoptosis [58,59], thereby suggesting that PARP hyperactivation in FE-PW is probably related to enhanced apoptotic cell death. Likewise, in vitro studies have demonstrated that PARP appears to suppress the differentiation of trophoblast cells [60], which could negatively affect placental functionality. Collectively, changes in the expression of NOX-1, NOX-2, iNOS and PARP support an increased exposure to OS in women who undergo a psychotic episode during pregnancy, although further efforts are warranted to establish the biological and clinical impacts of these alterations. In this sense, previous works have evidenced that a high maternal OS level corresponds to an even higher level of OS in the newborn with accelerated cellular senescence, which contributes to maternal and neonatal disorders [61]. In addition, a growing body of evidence relates increased OS in the placental tissue with foetal reprogramming and an increased risk of suffering from different pathological conditions in the childhood or in the adulthood, including neuropsychiatric disorders [15,62,63]. Further efforts are required to evaluate the middle- and long-term risk of suffering from a first episode of psychosis in the offspring.

## 5. Conclusions

For the first time, we showed that the placental tissue of FE-PW displays an augmented expression of OS markers (NOX-1, NOX-2, iNOS and PARP). These results can aid in explaining why an episode of psychosis during pregnancy has detrimental consequences for maternofoetal well-being. There is a need for future studies in this field to guide the proper clinical management of this psychiatric condition, also considering the middle- and long-term impact of suffering from a psychotic episode in pregnancy.

## Figures and Tables

**Table 1:** Clinical and demographic characteristics of women with a first psychotic episode during pregnancy and healthy controls. FE-PW = first psychotic episode during pregnancy; HC-PW = healthy control pregnant women.

	FE-PW (*n* = 22)	HC-PW (*n* = 20)
Median age (IQR), years	33.5 (21–42)	33.5 (25–39)
Median gestational age (IQR), weeks	40 (38–41)	40 (39–42)
C-section delivery, *n* (%)	3 (13.6)	2 (10.0)
Previous pregnancies, *n* (%)	8 (36.4)	9 (45.0)
Previous abortions, *n* (%)	1 (4.5)	2 (10.0)
Regular menstrual cycles, *n* (%)	17 (77.3)	16 (80.0)
PANSS Mean (SD)	Positive 18.8 (6.3)	-----
	Negative 25.7 (7.9)	

**Table 2:** Primers used for RT–qPCR: sequences and binding temperatures (Temp).

GENE	SEQUENCE Fwd (5′→3′)	SEQUENCE Rev (5′→3′)	Temp
TBP	TGCACAGGAGCCAAGAGTGAA	CACATCACAGCTCCCCACCA	60 °C
NOX-1	GTTTTACCGCTCCCAGCAGAA	GGATGCCATTCCAGGAGAGAG	55 °C
NOX-2	TCCGCATCGTTGGGGACTGGA	CCAAAGGGCCCATCAACCGCT	60 °C
iNOS	CCTTACGAGGCGAAGAAGGACAG	CAGTTTGAGAGAGGAGGCTCCG	61 °C
eNOS	AAGAGGAAGGAGTCCAGTAACACAGA	ACG AGC AAA GGC GCA GAA	60 °C
PARP	CCAGGATGAAGAGGCAGTGAAG	TTCTGAAGGTCGATCTCATACTCC	58 °C

Gene Accession number is available in the supplementary materials.

**Table 3:** Primary and secondary antibodies and their dilutions.

Antigen	Species	Dilution	Provider	Protocol Specifications
NOX 1	Rabbit polyclonal	1:250	Abcam (ab78016)	10 mM sodium citrate pH = 6 before incubation with blocking solution
NOX 2	Goat polyclonal	1:500	Abcam (ab111175)	0.1% Triton X-100 in PBS, 10 min, before incubation with blocking solution
iNOS	Rabbit polyclonal	1:350	Abcam (ab95866)	10 mM sodium citrate pH = 6 before incubation with blocking solution
eNOS	Rabbit polyclonal	1:50	Abcam (ab66127)	EDTA pH = 9 before incubation with blocking solution
PARP	Mouse monoclonal	1:1000	Abcam (ab110915)	10 mM sodium citrate pH = 6 before incubation with blocking solution
IgG (Rabbit)	Mouse	1:1000	Sigma–Aldrich (RG-96/B5283)	------
IgG (Goat)	Mouse	1:100	Sigma–Aldrich [GT-4/B3148]	------
IgG (Mouse)	Goat	1:300	Sigma–Aldrich (F2012/045K6072)	------

## Data Availability

The data used to support the findings of the present study are available from the corresponding author upon request.
